# Bacteriophage LDT325 enhances *Pseudomonas syringae* tolerance by improving antioxidant defense in tea plant [*Camellia sinensis* (L.) O. Kuntze]

**DOI:** 10.3389/fmicb.2024.1525040

**Published:** 2025-01-07

**Authors:** Li Liu, Anqi Huang, Hua Zhang, Yubao Li, Lei Wang

**Affiliations:** ^1^College of Agriculture and Biology, Liaocheng University, Liaocheng, China; ^2^School of Pharmaceutical Sciences and Food Engineering, Liaocheng University, Liaocheng, China; ^3^National Key Laboratory of Macromolecular Drug Development and Manufacturing, Liaocheng University, Liaocheng, China

**Keywords:** *Camellia sinensis*, *Pseudomonas syringae*, bacteriophage, physiological characters, antioxidant enzyme

## Abstract

Bud blight caused by *Pseudomonas syringae* is a serious disease affecting tea plants and causing severe damage to production output and quality. Phages play an important role in controlling the development of bacterial diseases in plants. Previous studies have shown that the tolerance of phage-treated tea plants to bud blight was notably greater compared with that of the control group. In the present study, we determined the effect of bacteriophage therapy on physiological and biochemical parameters of tea leaves. Transmission electron microscopy (TEM) was used to analyze the cellular structure of tea leaves, and bioinformatics was used to analyze the phage. Results revealed that bacteriophage treatment can enhance the expression of antioxidant enzyme genes (*CsSOD, CsCAT*, and *CsPOD*). The levels of osmotic adjustment compounds, including proline and soluble sugars, were also elevated, suggesting that bacteriophage enhances the osmotic adjustment capacity in tea plants. TEM analysis revealed that the integrity of the cell structure of the tea leaves treated with phage was notably better compared with that of the control group. Interestingly, we also observed that the phage lysed the animal pathogen *Salmonella* as well as the plant pathogen *P. syringae*. Using NCBI BLASTn to compare the entire genome with other nucleotide sequences, we found that the phage LDT325 exhibited cross-species characteristics that had not been previously reported. In summary, our findings demonstrate that bacteriophages can protect tea plants from damage caused by bacterial diseases by regulating antioxidant systems.

## 1 Introduction

The tea plant is a notable perennial evergreen crop that thrives in tropical and temperate regions (Hao et al., 2018). However, the warm and humid climate promotes the growth and transmission of pathogens that cause numerous plant diseases. Recently, there has been a significant increase in the occurrence of bacterial bud blight in tea plants throughout China. Diseases caused by *Pseudomonas syringae* are widespread and have affected the United States, Australia, and Korea (Tsuji and Takikawa, 2018). Tea bud blight primarily impacts the young buds and leaves. Tea plantations suffering from bud blight can experience a decline in productivity and quality (Khandan et al., 2013; Bartoli et al., 2015).

*P. syringae* causes a wide-range of bacterial diseases in plants worldwide. The emergence and spread of *P. syringae* in many tea-producing areas around the world have negatively impacted the sustainability of tea plants (Xin et al., 2018; Yang et al., 2023). *P. syringae* is highly aggressive and spreads rapidly among different plant varieties (Khandan et al., 2013; Bartoli et al., 2015). At present, the management of bud blight disease primarily depends on copper-based treatments and antibiotics; however, studies have shown that the protective effect of copper preparations significantly decreases after plants become infected with bacterial pathogens and improper use can lead to serious harm to the plants (Zhang et al., 2021). Although antibiotics can effectively reduce bacterial diseases, their overuse can promote bacterial resistance and residue accumulation in tea leaves (Batuman et al., 2024). Thus, the growing public consciousness of food safety has resulted in extensive recognition of the necessity of secure and efficient biological control methods for plant diseases.

Bacteriophage is a kind of virus that can specifically infect and destroy bacteria, and has the ability of self-replication. Unlike most antibiotics, phages generally demonstrate strong specificity for particular bacterial species or strains (Nawaz et al., 2023). Their ability to self-replicate enables them to be used as potent antimicrobial agents. Phages have been used not only for treating and preventing bacterial diseases in humans but also for managing plant diseases, detecting pathogens, and assessing food safety (Davidson et al., 2012; Lahlali et al., 2022). In addition, phages are also used to treat and prevent animal diseases, showing good application prospects in animal medicine and aquaculture. Bacteriophages significantly reduce their impact on the environment and non-target microorganisms. Thus, phages are considered more sustainable and safer compared with antibiotics (Buttimer et al., 2017).

Although significant advancements have been made in examining the role of phage in responses to plant bacterial diseases, little information regarding the effect of phage on bacterial disease tolerance in tea plants is available. In this study, we examined the regulatory mechanisms governing phage-mediated bacterial disease resistance in tea plants and evaluated the effect of phage on bacterial disease in tea plants. We determined the effect of phage treatment on various tea seedling factors during tea bud blight, including chlorophyll content, soluble sugar content, free proline (Pro) content, antioxidant enzyme activity, and the expression of the antioxidant defense system.

## 2 Materials and methods

### 2.1 Plant material and treatments

The tea variety used in this experiment was Longjing No. 43 (*Camellia sinensis*), which was grown in plastic boxes and cultured in acidic soil. The surface of the leaves was washed with sterile water and disinfected with 75% ethanol for 1 min. After the surface of the leaves was dried, four holes of equal distance were pierced in the middle of the leaves with sterile needles. The 40 μL of *P. syringae* (2 × 10^7^ CFU/mL) was applied to the surface of the leaves and distributed at four needle puncture sites for air drying. The 40 μL of phage suspension (2 × 10^7^ PFU/mL) was similarly applied to the upper surface of the leaves and spread across the same puncture sites. The treated leaves were covered with sterile cotton to protect the injured areas. For comparison, the control group was only infected with the pathogens (Aftab et al., 2022). The negative control was the sterile water group, in which 40 μL of sterile water was applied to the surface of the leaves and distributed at 4 needle puncture points for air drying. The treated leaves were cultured in a climate box at 25°C and 90% humidity for 3 days.

### 2.2 Sample collection and enzyme extraction

Leaf samples were collected to evaluate the activity of the antioxidant enzymes. The method of extracting the antioxidant enzymes was modified. Leaf samples (1 g) were ground using 5 mL of 50 mM phosphate buffer (pH 7.8). The resulting homogenate was centrifuged at 10,000 × *g* for 30 min at 4°C. The supernatants were immediately used to measure enzyme activity (Wang et al., 2024).

### 2.3 Superoxide dismutase (SOD) activity

A reaction mixture containing 130 mM methionine, 0.75 mM nitroblue tetrazolium, 0.1 mM ethylenediamine tetraacetic acid, and 0.05 M phosphate buffer (pH 7.8) was used to assess SOD activity. Briefly, 0.1 mL of the leaf extract supernatant was added to 3 mL of the reaction mixture. Then, 0.2 mL of a 0.02 mM riboflavin solution was added and the mixture was exposed to fluorescent light (4,000 lux) for 20 min to start the reaction. The complete reaction mixture without the enzyme extract was used as a control. The absorbance at 560 nm was used to calculate SOD activity.

### 2.4 Catalase (CAT) activity

CAT activity was determined using an ultraviolet spectrophotometer. The reaction solution was prepared with 30% hydrogen peroxide and 50 mM phosphate buffer (pH 7). Then, 0.1 mL of enzyme extract were added to the reaction mixture to start the reaction. The complete reaction mixture without the enzyme extract was used as a control. The absorbance at 240 nm was recorded every 30 s for 3 min.

### 2.5 Peroxidase (POD) activity

Peroxidase activity was determined by the guaiacol method. First, 50 mM phosphate buffer (pH 7) was added into a beaker followed by 1 mL of guaiacol; the mixture was heated and stirred. The resulting solution was cooled and mixed with 30% H_2_O_2_, and the leaf extract supernatant was mixed with the reaction solution. The control group was added to the same volume of 50 mM phosphate buffer (pH 7) solution without the enzyme solution. The absorbance was measured at 470 nm.

### 2.6 RNA extraction and quantitative real-time PCR

**Figure 1:**
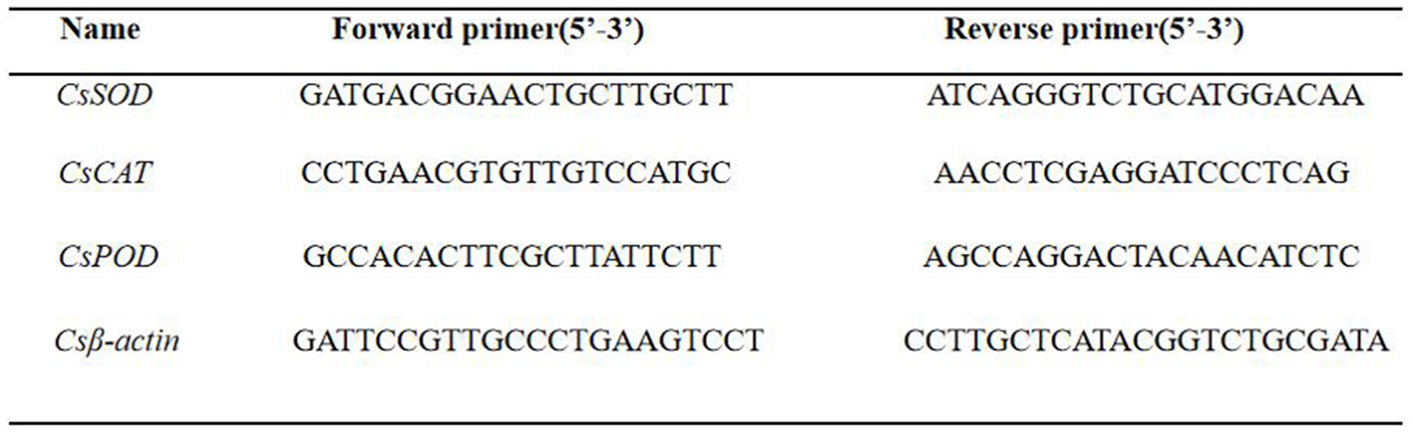
Primers used for gene expression analysis.

The leaf-damaged part was ground into a fine powder in the presence of liquid nitrogen with a high-pressure sterilized mortar and pestle and stored at −80°C (Aftab et al., 2022). The RNA Isolator Total RNA Extraction Reagent (Vazyme, Nanjing, China) was used to extract total RNA from the tea tree tissues, which was reverse-transcribed into cDNA using a reverse transcriptase (ReverTraAce-α, Toyobo Co.). The internal reference gene was Csβ-actin. Real-time qPCR was done using an Applied Biosystems 7500 Real-Time PCR system (Thermo Fisher Scientific, USA). The amplification program was as follows: 95°C for 30 s; 40 cycles at 95°C for 5 s, and annealing at 60°C for 30 s; 95°C for 15 s, 60°C for 1 min, and 95°C for 15 s. The relative expression was calculated using the 2^−ΔΔCt^ method (Wang et al., 2023). The primer sequences are listed in Figure 1. The expression levels were assessed using three replicates.

### 2.7 Determination of proline (pro) content in tea leaves

The proline content was measured using a slightly altered method. Leaf samples (1 g) were ground in 10 mL of 3% sulfosalicylic acid and the mixture was centrifuged at 10,000 g for 10 min. Next, 2 mL of supernatant was mixed with 2 mL of glacial acetic acid and 2 mL of ninhydrin reagent, then heated in a water bath for 40 min. After cooling to room temperature, 5 mL of toluene was added and the mixture was shaken (Freitas et al., 2019). After the solution formed a layer, the upper solution was removed and the absorbance at 520 nm was measured.

### 2.8 Determination of soluble sugar content and chlorophyll content in tea leaves

The anthrone method was used to determine the soluble sugar content. A small amount of distilled water and 1.0 g of leaves were added to grind and homogenize. After heating the mixture in a water bath for 30 min, it was cooled and filtered into a 100 mL volumetric flask. After adding 5 mL of distilled water, the residue was extracted with boiling water and filtered into a volumetric flask to a constant volume. The extract (0.5 mL) was transferred to a 25 mL test tube and 5.0 mL of concentrated sulfuric acid and 0.5 mL of anthrone-ethyl acetate reagent were added. After thorough mixing, the mixture was boiled for 1 min (Luo and Huang, 2011). After cooling, the absorbance value was measured at 630 nm.

The chlorophyll content was measured using a spectrophotometer. The leaves (1 g) were mixed with 2.5 mL of 80% acetone solution and a small amount of quartz sand and ground. Next, 10 mL of acetone solution was added and grinding was continued until the sample appeared white. The sample was filtered into a volumetric flask and 80% acetone solution was added to a constant volume. The absorbance was measured at 652 nm (Pérez-Patricio et al., 2018).

### 2.9 Transmission electron microscopy (TEM)

The tea leaves were immersed in 2.5% glutaraldehyde for 12 h, then removed and rinsed with 0.1 M phosphate buffer (pH 7.0) 3–5 times. Each rinse lasted 15 min. Then, 1% osmotic acid solution was added and incubated for 1–2 h. To remove the excess osmotic acid solution, the sample was rinsed with the same concentration of phosphate buffer solution (pH 7.0) 3 times for 15 min each. The samples were dehydrated in a series of ethanol washes, with the concentration increasing at each step (50%, 70%, 80%, 90%, and 95%) for 15 min per step. Then, they were rinsed in 100% ethanol for 20 min and 100% acetone for another 20 min. The specimens were immersed in a 3:1 embedding agent and acetone solution for 180 min. The samples were embedded and ultra-thin sections (70–90 nm) were cut using a Leica ecaEMUC7 ultra-thin sectioning machine. The sections were mounted onto grids and stained with saturated aqueous uranyl acetate and leaf citrate for TEM (Hitachi, H-7500) (Li et al., 2014). The sterile water group, the treatment group and the control group were treated in the same way.

### 2.10 Genome sequencing and analysis of vB_PsS_LDT325

A virus extraction kit was used to extract vB_PsS_LDT325 phage nucleic acid. The phage genome samples were submitted to the BIOZERON company for sequencing and the host bacterial sequences were filtered out to obtain a valid phage sequence. The sequencing results were subjected to quality control using FastQC on the raw data. Genome assembly was done using Unicycle, prediction of the genome ORFs using GeneMarkS, and alignment and annotation of functional proteins using GenBank. BLAST searches against the NCBI database were conducted for sequence similarity analyses. Putative virulence factors were screened using the Virulence Factor Database (http://www.mgc.ac.cn/cgi-bin/VFs/v5/main.cgi) and antibiotic resistance genes were screened by the Comprehensive Antibiotic Resistance Database (https://card.mcmaster.ca/analyze/rgi) (Jia et al., 2016; Liu et al., 2019). For genome visualization, the Proksee Server (https://proksee.ca/) and Easyfig_2.2.5_win were selected. Evolutionary trees were constructed using the ClustalW program in MEGA (Kumar et al., 2018).

### 2.11 Double-layer plate method was used to verify whether phage LDT325 lyses Salmonella

The double-layer plate method was used to determine whether the phage lyses Salmonella. The 200 μL Salmonella solution (2 × 10^7^ CFU/mL) was poured into 5 mL LB semi-solid agar medium [LB containing 0.4% (w/v) agar] at 55°C, and then immediately poured into the plate of LB solid agar medium [LB containing 1.5% (w/v) agar] to prepare a double-layer plate. Approximately 10 μL of phage filtrate was added to the solidified semi-solid LB plate and cultured at 37°C for 12 h to observe whether there were transparent areas or plaques at the inoculation site. Purification: A single plaque was taken with a sterile gunhead, placed in SM buffer for 12 h, and filtered with 0.22 μm filter membrane. After filtration, the filtrate and Salmonella were taken to prepare a double-layer plate again, and a single plaque was taken for purification for 4 times to obtain a purified phage (Cao et al., 2022).

### 2.12 Statistical analysis

Data were analyzed using GraphPad Prism 8.0.2, specifically employing one-way analysis of variance. At least three independent replicates were performed under identical conditions, and data were presented as the mean ± standard deviation. Statistical significance was assessed based on *P*-value, and *P*-values of < 0.05 were considered to indicate statistical significance.

## 3 Results

### 3.1 Effect of phage on antioxidant enzyme activity in tea plant leaves

**Figure 2:**
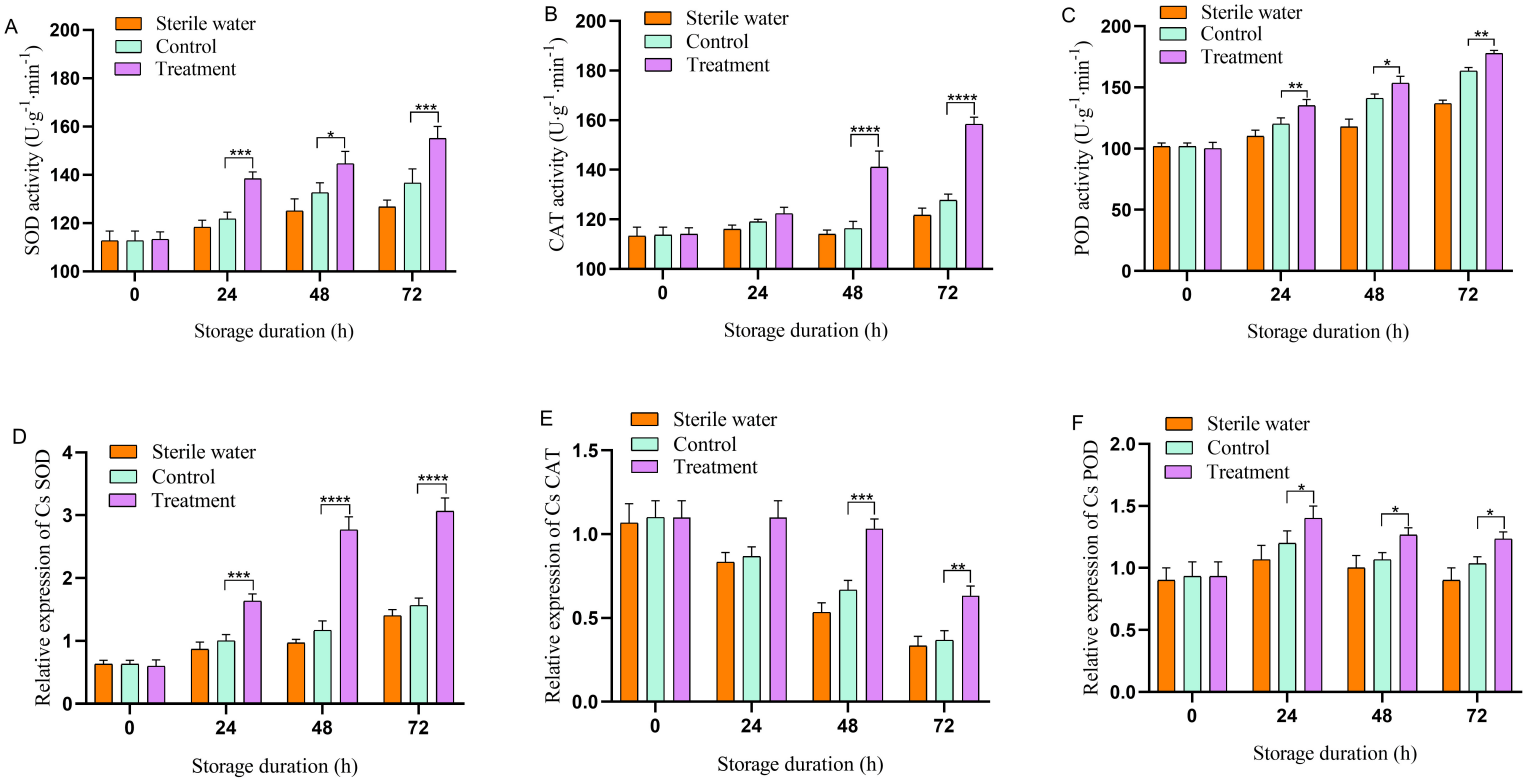
Effects of phage on antioxidant enzyme activity and gene expression in tea at designated time. **(A)** Superoxide dismutase (SOD), **(B)** catalase (CAT), **(C)** peroxidase (POD), **(D)** CsSOD, **(E)** CsCAT, **(F)** CsPOD. Vertical bars indicate the standard error of the mean values. Asterisks imply statistically significant differences (*p* < 0.05) between the control and phage treated group in the same day.

Antioxidant enzyme activity in the phage-treated leaves exhibited an upward trend. Inoculation of sterile water did not affect the antioxidant enzyme activity of tea leaves. The antioxidant enzyme activities of SOD, CAT, and POD in the leaves of the control- and phage-treated plants did not change significantly at the initial stage (Figures 2A–C). At 24–72 h, the antioxidant enzyme activity of phage-treated leaves was higher compared with that of the control group. At 24 and 72 h, SOD activity in the phage-treated group was significantly higher compared with that in the control group (13.11% and 11.11% higher, respectively). At 48 and 72 h, CAT activity in the phage-treated group was higher compared with that in the control group (24.35% and 24.03% higher, respectively). At 24 and 72 h, POD activity in the phage-treated group was significantly higher compared with that in the control group (8.33% and 9.38% higher, respectively). The results indicate that phage can enhance the activity of antioxidant enzymes.

### 3.2 Effect of phage treatment on the expression of antioxidant enzymes in tea plants

As shown in Figures 2D–F, inoculation with sterile water did not affect the relative expression of CsSOD, CsCAT, and CsPOD in tea leaves. The relative expression of *CsSOD, CsCAT*, and *CsPOD* in the leaves of the control and phage-treated plants did not change significantly at the initial stage; however, the relative expression of *CsSOD* in phage-treated leaves was significantly higher compared with that in the control at 24–72 h. At 48–72 h, the relative expression of CsCAT in the leaves of the phage-treated group was higher compared with that of the control. The relative expression of CsPOD in the control and phage-treated leaves exhibited a trend of increasing first and then decreasing; however, the relative expression of CsPOD in the phage-treated leaves was significantly higher compared with that in control throughout the experiment, reaching a peak at 24 h. These results indicate that phage treatment significantly increases the expression of antioxidant enzyme-related genes in tea leaves.

### 3.3 Effects of phage on the pro content, soluble sugar, and chlorophyll content in tea plants

**Figure 3:**
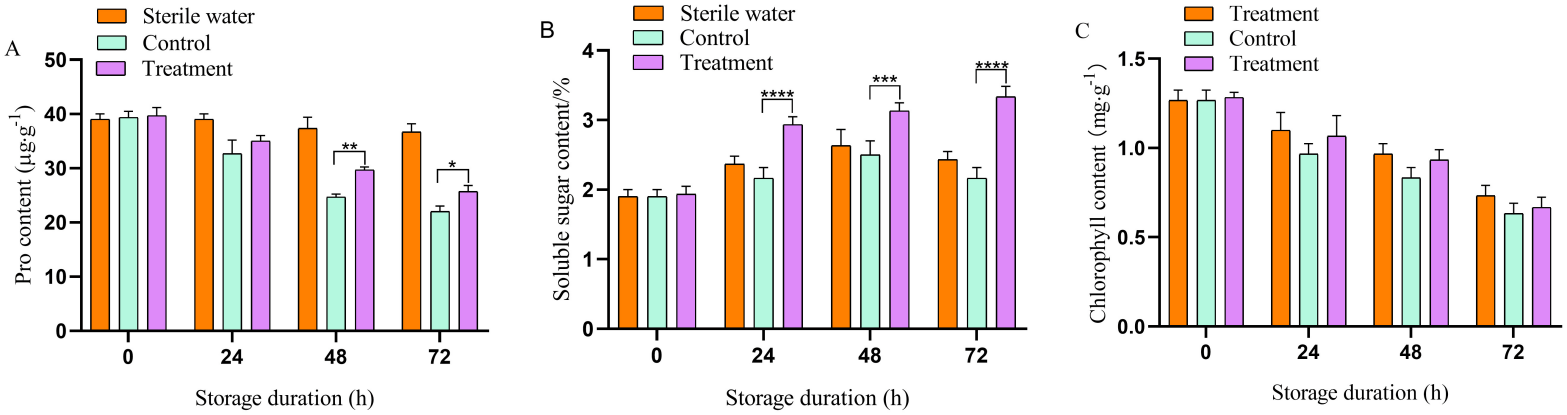
Phage affecting on physiological parameters of tea leaves. **(A)** Proline content, **(B)** soluble sugar content, **(C)** chlorophyll content. Vertical bars indicate the standard error of the mean values. Asterisks imply statistically significant differences (*p* < 0.05) between the control and phage treated group in the same day.

Inoculation of sterile water did not affect the proline content, soluble sugar content, and chlorophyll content in tea leaves. At 48 h, compared with the control group, the proline content in the phage-treated tea increased by 20.83% (Figure 3A). At 72 h, soluble sugar levels in tea leaves treated with phage increased by 45.11%, compared with that in the control group (Figure 3B). Furthermore, during the whole experiment, the soluble sugar and proline content in phage-treated tea leaves remained higher than that in the control group. Chlorophyll in the tea plants was reduced with an increase period of bacterial disease stress and phage treatment slowed the decline of chlorophyll in the leaves. The chlorophyll content in the tea leaves treated with phage consistently exceeded that of the control group (Figure 3C). At 72 h, the chlorophyll content of the phage group was significantly higher compared with that of the pathogen-treated group (16.67% higher).

### 3.4 Transmission electron microscope of tea leaves

**Figure 4:**
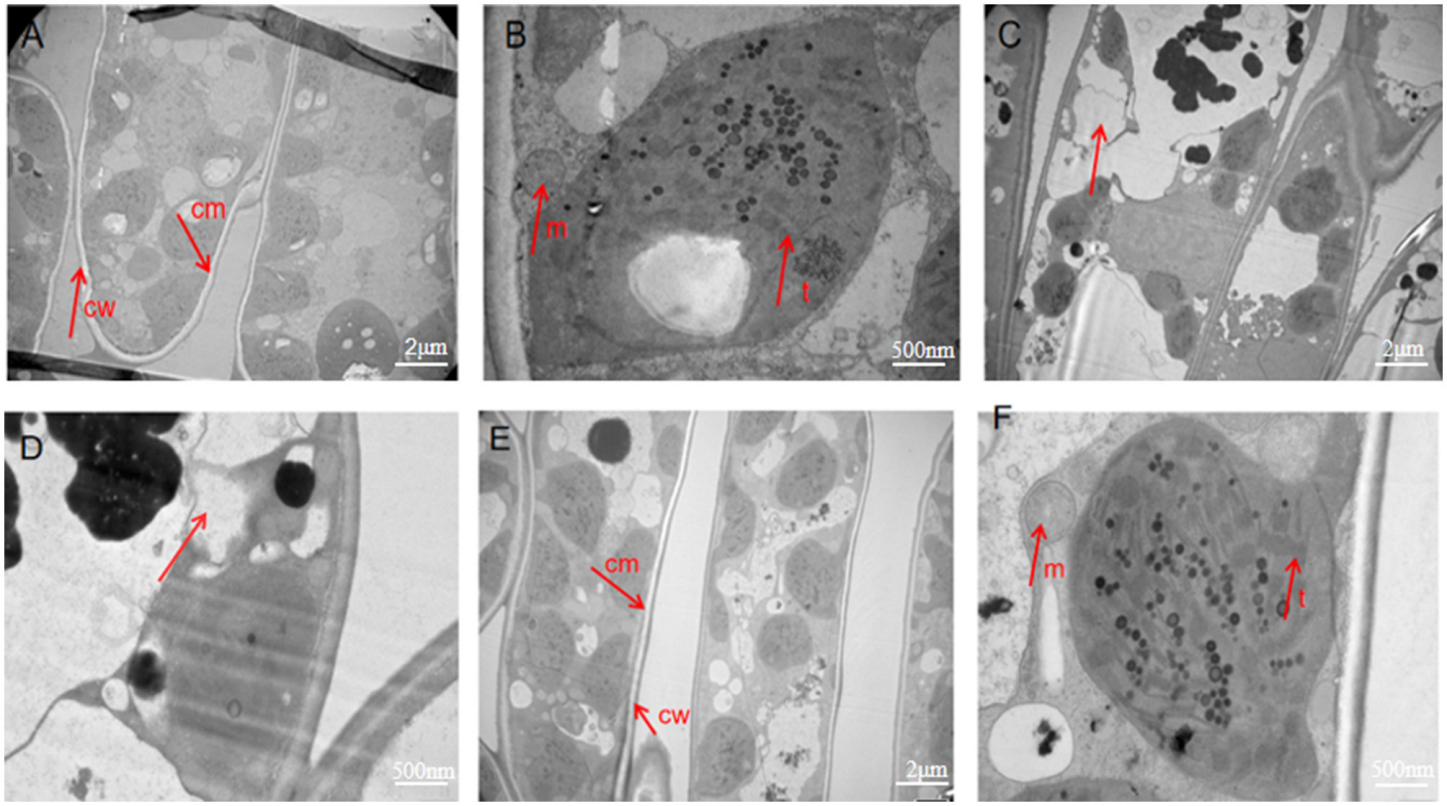
Transmission electron microscopy images of ultrathin sections of tea leaves. **(A, B)** Transmission electron microscopy images of ultrathin sections of tea leaves in sterile water group. **(A)** Observed at 1,000×. **(B)** Observed at 5,000×. **(C, D)**Transmission electron microscopy images of ultrathin sections of tea leaves in the control group. **(C)** Observed at 1,000×. **(D)** Observed at 5,000×. **(E, F)** Transmission electron microscopy images of ultrathin sections of tea leaves in phage treatment group. **(E)** Observed at 1,000×. **(F)** Observed at 5,000×. cw, cell wall; cm, cell membrane; m, mitochondria; t, thylakoid.

Cell morphology was observed by TEM. Image analysis of the sterile water group showed that the cell wall and cell membrane were clear, and there were complete cell structures, such as mitochondria and thylakoids (Figures 4A, B). These characteristics were the same as the internal morphological structure of healthy leaves. In contrast, the images of the control group infected with *Ps* exhibited cell lysis and a lack of cell structure, indicating necrosis (Figures 4C, D). The images of the phage-treated group showed clear cell structures, including a cell wall, mitochondria, thylakoid, and cell membrane (Figures 4E, F). This indicates that phage LDT325 effectively inhibits *P. syringae*.

### 3.5 Whole genomic and phylogenetic analysis of vB_PsS_LDT325

**Figure 5:**
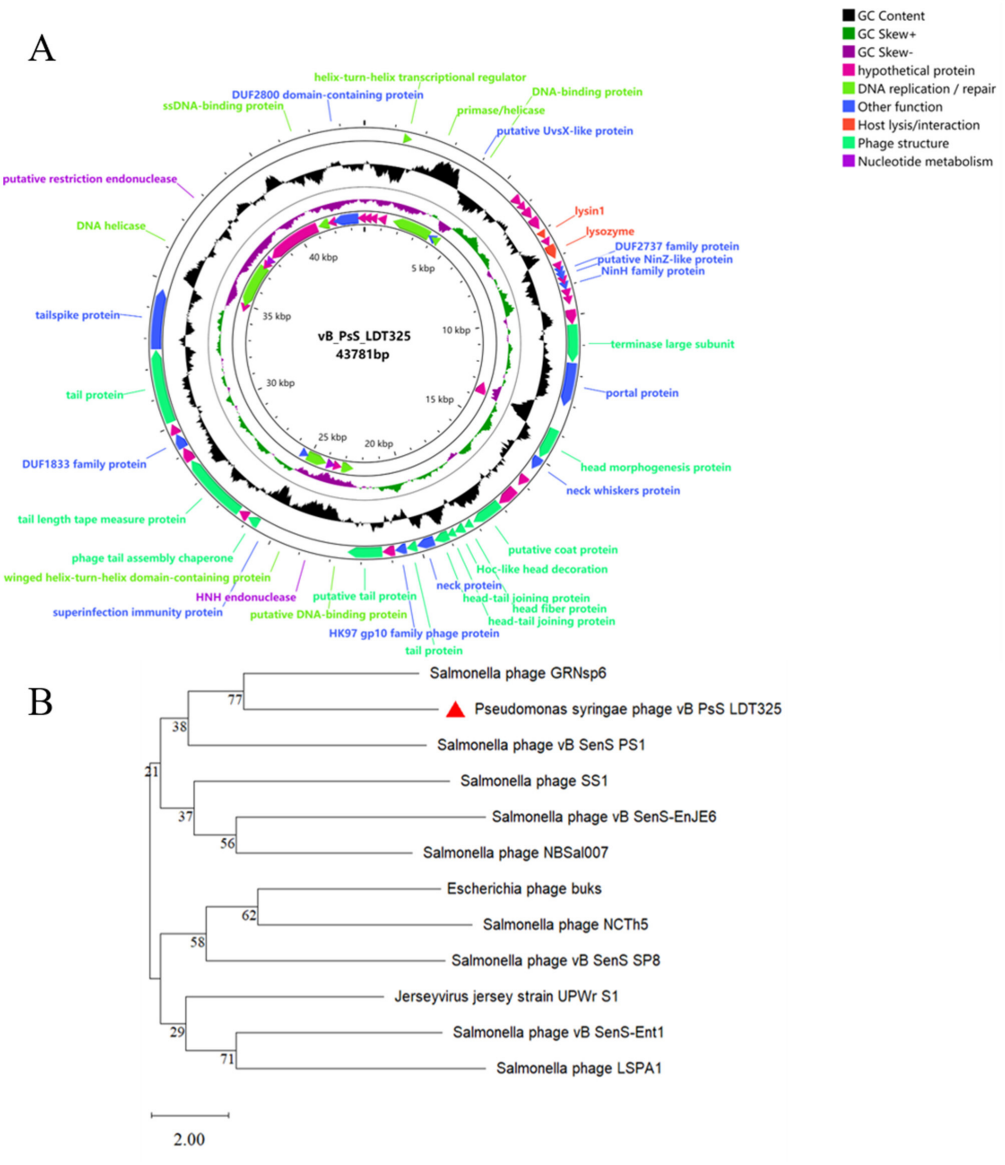
Genomic analysis. **(A)** Genome map of phage vB_PsS_LDT325, the genome of phage vB_PsS_LDT325 depicted in the circular. These arrows represent 61 CDS. In addition, the map shows GC skew and content about the genome. **(B)** An expanded view of the region of the tree containing the most closely related phages. The location of phage LDT325 is indicated in the red triangle.

The sequencing results indicate that vB_PsS_LDT325 is a long-tailed bacteriophage, which is in consistent with the findings from previous electron microscopy studies. Phage LDT325 has a length of 43,781 bp with a G+C% content of 48.82% (Figure 5A). The entire genomic sequence was subjected to an NCBI BLASTn analysis and the results indicated that it had high similarity with the *Salmonella* phage GRNsp6 (ON526838.1, query coverage 92%, nucleotide homology 94.98%). The complete genomic sequence and annotation information of the phage were submitted to GenBank under accession number PP389045. In addition, a phylogenetic tree was created using the amino acid sequence of the major capsid protein of the vB_PsS_LDT325 bacteriophage. The evolutionary status of the phage LDT325 was also evaluated by phylogenetic analysis to select the 11 existing phages from the database, including 9 *Salmonella* strains, 1 *Escherichia*, and 1 Jersey virus phage. Phylogenetic tree analysis revealed that the LDT325 phage was clustered on a distinct branch and its closest relative was phage GRNsp6 (Figure 5B). Phage LDT325 contains 61 CDS, including 4 DNA replication/repair functional proteins (DNA ligase, DNA polymerase, DNA primer enzyme, DNA helicase, DNA polymerase, RNA polymerase, integrated host factor), and 4 nucleotide metabolism proteins (ribonuclease, recombinant endonuclease, recombinant exonuclease), 15 phage structural proteins (main tail fiber protein, bottom plate protein, tail tube protein, tail sheath protein, main capsid protein, major termination enzyme subunit, gate protein), 1 host lysis/interaction protein (bacteriolysin, hole protein), and 11 other function proteins. The remainder are annotated as hypothetical proteins (Supplementary Table S1). The entire genome was uploaded to the Virulence Factor Database and the Comprehensive Antibiotic Resistance Database; no bacterial toxin genes or antibiotic resistance genes were detected. Additionally, lysogenic-related genes, such as integrase, recombinase, cleavage enzyme, and inhibitory enzyme, were not found.

### 3.6 Comparative genomics of phage vB_PsS_LDT325

**Figure 6:**
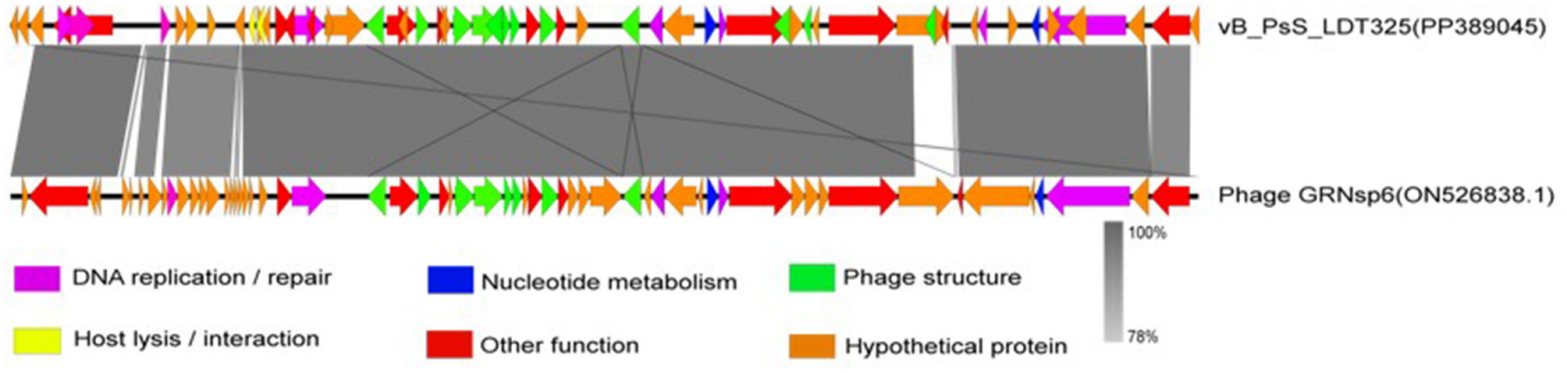
Genome structure. Arrows indicate the direction of transcription for predicted ORFs. ORFs with different functions are shown in different colors.

Phage vB_PsS_LDT325 was subjected to a multiple genomic comparison with the phage of the genus “*Salmonella* phage GRNsp6.” The results indicated that phage vB_PsS_LDT325 exhibited an extremely high protein homology (>78%). The homologous protein modules primarily included DNA replication/repair, nucleotide metabolism, and phage structure, particularly in terms of phage structure and DNA replication/repair (Figure 6).

### 3.7 Phage vB_PsS_LDT325 lyses Salmonella

**Figure 7:**
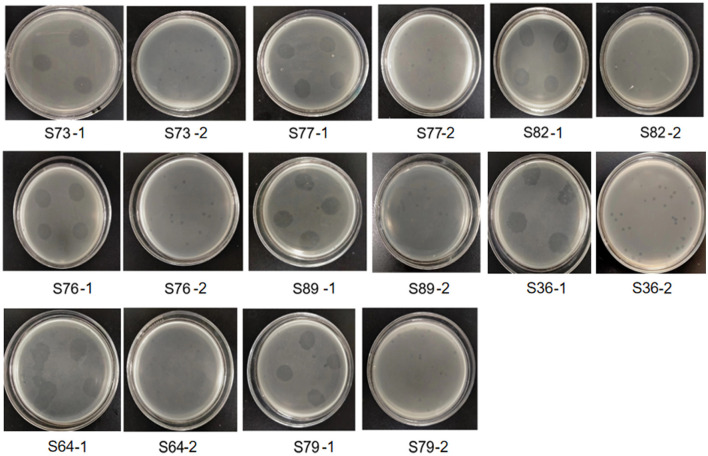
Several animal pathogenic Salmonella strains lysed by phage LDT325. The number below the picture is the number of Salmonella. Image S73-1 showed that after 12 h of culture, the phage suspension showed a clear cleavage area on the double-layer plate covered with Salmonella suspension numbered S73, showing the lysing bacteria. Picture S73-2 was further purified from the clear cleavage region of phage in picture S73-1. The same below.

The results showed that there were clear and bright plaques on the double-layer agar plate. After 4 times of purification, plaque with uniform size and morphology could be observed on the double-layer plate (Figure 7). Therefore, we isolated a phage that can lyse both plant pathogen *Pseudomonas syringae* and animal pathogen Salmonella.

## 4 Discussion

*P. syringae* can infect many economically important plants, causing the spread of plant diseases and serious economic losses. Because of the increased resistance of plant pathogens to widely used copper-based fungicides and antibiotics, phages have become an alternative method of biological control owing to their strong specificity and self-replication (Nachimuthu et al., 2021). Bacteriophages are capable of specifically infecting bacteria and can more effectively remove pathogens without affecting normal bacterial populations. Phage infection can activate the host immune response, thereby enhancing the host tolerance to bacterial infection. The interaction between phages, pathogens and beneficial bacteria is a complex and important ecosystem. Bacteriophages specifically infect specific bacterial hosts, and the enzymes released by phages during the lysis phase can directly dissolve the bacterial cell wall, leading to the death of pathogens. Bacteriophages also help maintain the balance of beneficial bacteria. Beneficial bacteria inhibit the growth of pathogens by competing for nutrients, producing antimicrobial substances and changing the microenvironment (Fernández et al., 2019). There are few reports to date on biological control methods related to the prevention and treatment of tea bud blight (Gu et al., 2021; Kim et al., 2019; Wang et al., 2020). Moreover, there are only a limited number of studies showing the effectiveness of phage inhibition on *P. syringae* infection by analyzing the expression of antioxidant enzymes and related genes or through TEM of tea leaves.

The accumulation of reactive oxygen species causes oxidative damage to plants; therefore, they have evolved a set of antioxidant systems, including antioxidant enzymes (SOD, POD, CAT) (Hasanuzzaman et al., 2020). Antioxidant enzymes can maintain the balance between the production and scavenging of reactive oxygen species and free radicals to maintain homeostasis. During pathogen infection, plant cells often experience oxidative stress, resulting in damage to cell membranes, proteins, and DNA. Increasing the activity of antioxidant enzymes can reduce these damages and help plants maintain normal physiological functions and growth. Plants may establish a long-term effective defense mechanism by increasing the activity of phage-induced antioxidant enzymes. The phage itself can directly infect and lyse *Pseudomonas syringae*, reducing the number of pathogens, thereby reducing the risk of plant infection. At the same time, phage infection may stimulate the immune response of plant cells and improve disease resistance (Alkadi, 2020). The increase of antioxidant enzyme activity induced by phage and the enhancement of plant immune response promote each other, and jointly improve the resistance to *Pseudomonas syringae*. Peroxidase (POD) is a key enzyme in the enzymatic defense system of plants under stress conditions. For example, balancing zinc nutrition can improve the antioxidant activity of lily flowers to extend storage time (Shaheen et al., 2015). We analyzed the effects of phages on the expression of antioxidant (*CsSOD, CsCAT*, and *CsPOD*). The results indicated that phage significantly increased the transcription levels of these genes. Another study demonstrated that treatment with spermidine increased the expression of antioxidant genes and the activity of antioxidant enzymes, while decreasing reactive oxygen species production in alfalfa exposed to salt stress (Lou et al., 2018). Consistent with these findings, we demonstrated that phage treatment enhances the activities of key antioxidant enzymes under abiotic stress by increasing the expression levels of their associated genes, thus improving disease resistance in tea plants. The sterile water group had no effect on antioxidase activity and related gene expression, proline content, soluble sugar content and chlorophyll content.

Proline and soluble sugar are important osmotic adjustment substances in plants. They are one of the indicators that reflect the level of plant stress resistance. Furthermore, the application of phage upregulated chlorophyll and soluble sugar levels in tea leaves, which was consistent with studies showing that COS in wheat enhanced the accumulation of these components (Naz et al., 2021). Chlorophyll is the main substance required for photosynthesis of plants (Baltazar et al., 2021). Soluble sugar is used for plant metabolism and is an important energy source for plant growth and development (Afzal et al., 2021). During stress, proline levels in plants increases significantly (Qamar et al., 2015). Proline also activates the expression of plant defense genes, enhances the function of the immune system, and improves the resistance of plants to pathogens. During abiotic stress, proline levels increase as a result of significant protein degradation. In the present study, the notable rise in proline levels in tea leaves is indicative of disease resistance. The use of phages in tea cultivation enhances the growth of tea plants and boosts production yields. Therefore, phages positively influence the physiological responses of tea plants by promoting their growth and development.

The cell membrane has various physiological functions, including the role of a barrier to maintain a stable intracellular environment (Stewart et al., 2018). It controls the exchange of internal and external substances of cells and regulates the life activities of cells. We showed that the integrity of cells in the control group was compromised, which was confirmed by TEM. Specifically, the cell membrane in the control group was completely ruptured, which resulted in the collapse of intracellular structures. This indicates that pathogens destroy the cell membrane structure, interfere with cell membrane permeability, and induce apoptosis. The images of the phage treatment group showed clear cell structures, including a cell wall, mitochondria, thylakoid, and a cell membrane. Thus, phage may severely impede the growth of *Ps*, which has a marked impact on its propagation. This indicates that phage LDT325 effectively inhibits *P. syringae*. Whole genome analysis of vB_PsS_LDT325 phage provides a theoretical basis for its effect on *P. syringae*. The phage genome obtained in this study was 43,781 bp in length with a GC content of 48.82% and 61 ORFs. The GC content of phage LDT325 was higher compared with that of *P. syringae* phages, such as KIL1 and KIL2, with an average GC content of 44.8% (Rombouts et al., 2016). The host specificity of bacteriophages comes from the specific recognition of the host surface receptors. The tail filament proteins of most bacteriophages are responsible for the specific recognition of host receptors. Phage LDT325 has four ORFs encoding the family proteins, ORF16, ORF19, ORF37, and ORF49. A group or several groups of proteins with similar amino acid sequences are known as protein families and evidence of the evolution of multiple species can be obtained by analyzing protein family members. Phage LDT325 contains a gene that encodes the portal protein ORF24. The function of the portal protein is similar to that of a DNA sensor, which can couple genome packaging with icosahedral capsid maturation (Lokareddy et al., 2017). The tail proteins, ORF36, ORF51, and ORF47, comprise the tail phage, which indicates that the phage LDT325 belongs to the tail phage (Chibani et al., 2019). Phage LDT325 expresses two genes encoding endolysin, ORF12 and ORF14. Compared with traditional broad-spectrum antibiotics, the main advantage of endolysin is its high specificity; moreover, it does not kill beneficial bacteria (Gontijo et al., 2021; Rahman et al., 2021). Therefore, as a medicinal bacteriostatic agent, phages have obvious advantages compared with traditional antibiotics. Interestingly, we found that the phage LDT325 lysed the animal pathogen *Salmonella* as well as the plant pathogen *P. syringae*. This phenomenon has not been reported to date. In addition, bacterial toxin genes, antibiotic resistance genes, integrase, recombinase, cleavage enzymes, and inhibitory enzymes related to lysogenic genes were not found in the genome of vB_PsS_LDT325, and indicates its safety as an antibacterial agent for potential clinical application. Whole genome phylogenetic tree construction, linear analysis of amino acid sequences, and the development of a tree based on the major capsid protein indicate that vB_PsS_LDT325 has a close relationship with other bacteriophages and demonstrates the genetic diversity among bacteriophages.

In conclusion, we demonstrated that phages have multiple roles in preventing *P. syringae* from infecting tea plants, such as inducing antioxidant enzyme activity, enhancing plant resistance to pathogenic bacteria, destroying the cellular structure of *P. syringae*, and inhibiting the expansion of *P. syringae*. Our findings demonstrate that the phage LDT325 has numerous beneficial effects on tea plants, thus indicating the potential for application in the prevention and control of tea leaf blight.

## Data Availability

The datasets presented in this study can be found in online repositories. The names of the repository/repositories and accession number(s) can be found below: NCBI.

## Supplementary material

Supplementary Table S1 Genomic annotation of *Pseudomonas syringae* phage *vB_PsS_LDT325*.
